# Seasonal and Interannual Variability in the Insect Pest Damage and Beneficial Insect Populations Across Apple Orchards of Different Ages

**DOI:** 10.3390/insects17030341

**Published:** 2026-03-20

**Authors:** Kornél Komáromi, Mihály Zalai, Ágnes Kukorellyné Szénási, Zita Dorner

**Affiliations:** Department of Integrated Plant Protection, Plant Protection Institute, Hungarian University of Agriculture and Life Sciences, 2100 Gödöllő, Hungaryzalai.mihaly@uni-mate.hu (M.Z.);

**Keywords:** *Cydia pomonella*, *Adoxophyes orana*, *Anthonomus pomorum*, *Phyllonorycter* spp., natural enemies, ecosystem service, IPM

## Abstract

Apple is one of the most important fruits worldwide; in addition, it constitutes nearly 60% of Hungary’s total fruit production. Presently, the challenge in pest management is not only the decreased range of pesticides, but also the deeper knowledge of natural enemies occurring in fruit plantations, and in their neighborhood. Our objective was to study how season, year, orchard structure, and varieties influence the occurrence of insect pest damage and beneficial insects in apple. We also investigated the strength of correlations between pest damage and natural enemies, as well as among the different natural enemy taxa. The experiment was conducted in three apple orchards, and the damage of insect pests and the number of natural enemies were monitored across ten trees/varieties/sectors/dates. All sites were managed according to integrated pest management (IPM) guidelines, and no unmanaged or untreated control plots were included. Significant differences were observed among orchards and years for all taxa, except *Cydia pomonella* (L.) among orchards and *Orius* spp. among years. Pest populations are primarily driven by seasonal and climatic factors, while beneficial insects are shaped more by local habitat features and orchard structure. No effect of the varieties on insect damage could be detected, whereas the abundance of beneficial organisms differed significantly among varieties in more cases. The weak correlations between pests and their natural enemies suggest that the presence and activity of predators depend not only on pest abundance but also on other interrelated ecological factors in intensive apple orchards. The damage caused by *C. pomonella* could also be kept at a low level in all orchards when appropriate pest management technology is applied.

## Simple Summary

Insect pest management in apple orchards increasingly relies on biological control strategies to sustainably suppress pest populations while minimizing environmental and chemical inputs. The study objectives were to determine how season, year, orchard structure, and apple varieties influence the occurrence of insect pests and beneficial insects, and to assess the strength of correlations between pest damage and natural enemies, and among natural enemy taxa. Experiments were performed in two consecutive years in three different-aged orchards, and on 11 varieties, where insect damage and the number of natural enemies were observed. All sites were managed according to integrated pest management (IPM) guidelines, and no unmanaged or untreated control plots were included. The main pests were the codling moth, the summer fruit tortrix, the leafminer moth, and the apple blossom weevil; however, their damage could be maintained at low levels. Among natural enemies, aphidophagous arthropods dominated. Pest populations are primarily driven by seasonal and climatic factors, while beneficial insects are shaped more by local habitat features and orchard structure. Our findings should be of broad interest to entomologists, agroecologists, and practitioners of sustainable fruit production.

## 1. Introduction

Apple belongs to a very major group of fruit worldwide. Global production of apples has been consistently increasing. About ten years ago, it was somewhat over 80 million tons; more recently, it is estimated as substantially exceeding 90 million tons per year [1]. However, following recent USDA data, marketable fresh fruit production has diminished by 0.32 million tons [2]. The great significance of apple production in Hungary is shown by the fact that Hungary is the fifth most important apple-producing country in Europe. Remarkably, apple constitutes nearly 60% of Hungary’s total fruit production [3]. The producers prefer those apple varieties that can be easily stored for as long as 6 to 8 months in the warehouse. Examples of this producer policy are in Hungary: Jonathan, Gala, Pinova, Gloster, Jonagold, and Idared [4]. In addition, during the recent decades, a significant reason for the choice of the apple varieties proposed is the fruit’s disease resistance capacity [5].

At present, the greatest challenge both in Hungary and in most other countries is to develop such sustainable agricultural practices that produce, at the same time, healthier food and leave a reduced carbon footprint [6,7]. This type of agricultural practice can only be realized by a constantly increased focus on research on ecosystem services [8]. To understand the significance of ecosystem services, the regulating factors of the environment should be known, in this case, the relation between semi-natural habitats and orchards, as well as between insect pests and their natural enemies. Semi-natural habitats are essential parts of agroecosystems and can maintain ecosystem services such as pollination, and biological control [9].

Sustainable pest management in fruit production faces increasing challenges due to climate change and reduced pesticide availability [10]. Insect pest management in apple orchards increasingly relies on biological control strategies to sustainably suppress pest populations while minimizing environmental and chemical inputs [11].

Porcel et al. (2018) [12] reported that a more complex habitat structure enhances the diversity of natural enemies and improves biological control. In addition, Simon et al. (2011) [13] emphasized that orchard biodiversity plays a key role in sustaining beneficial organisms and promoting sustainable pest control.

Insect pest and natural enemy taxa regularly occurring in Hungarian apple orchards [14,15], as well as in the orchards investigated by the authors, are presented as follows. Codling moth, *Cydia pomonella* (L.) (Lepidoptera: Tortricidae), is one of the most important arthropod pests of apple; it is the cause of serious yield loss in apple worldwide [16]. The damage is attributed to young larvae inside the fruit, which reduces the harvestable quantity and negatively affects the storage ability [17]. Another substantial insect pest in the European apple orchards is the summer fruit tortrix, *Adoxophyes orana* Hbn. (Lepidoptera: Tortricidae). Its younger larvae chew the leaves, and the older ones the fruits [18]. Leafminer species *Phyllonorycter* spp. (Lepidoptera: Gracillariidae) create their mines inside the leaf, and their damage negatively affects photosynthesis, while water-use efficiency positively affects it [19]. In European apple orchards, the apple blossom weevil, *Anthonomus pomorum* L. (Coleoptera: Curculionidae), is a significant pest during the early vegetation period because it attacks flower buttons, thereby decreasing yield [20].

The common flowerbug, *Anthocoris nemorum* (L.) (Hemiptera: Anthocoridae), is a polyphagous predatory bug that is dominant in spring in apple orchards. Feeding on aphids, psyllids, young Lepidoptera larvae, and mites, this species contributes to the decrease in arthropod pest numbers [21]. Anthocorid bugs, *Orius* spp. (Hemiptera: Anthocoridae), are effective predators of phytophagous mites in apple plantations [22]. European earwig *Forficula auricularia* L. (Dermaptera: Forficulidae) can consume both arthropods and plant parts. In orchards, it is mainly considered a beneficial insect species [23]. Aphid midge, *Aphidoletes aphidimyza* (Rond.) (Diptera: Cecidomyiidae), is specialized to aphids; in greenhouses, it is often used as a biological agent [24]. Hoverflies (Diptera: Syrphidae) offer a double benefit for the agroecosystem: imagoes are pollinators, and larvae are predators of aphids; the larvae can be used as biological control agents [25]. Snakeflies (Raphidioptera: Raphidiidae) occur primarily in arboreal habitats; their adults and larvae are both predators. The imagoes feed mostly on aphids, other Sternorrhyncha, and also on pollen. The larvae can prey on different stages of arthropods, for instance, on Lepidoptera and Coleoptera eggs and larvae [26]. Green lacewing (Neuroptera: Chrysopidae) larvae play an important role in aphid regulation. The imagoes consume nectar, pollen, and honeydew; furthermore, depending on the species, they also prey on aphids and other insects [27]. Both the larvae and the adults of the seven-spot ladybird, *Coccinella septempunctata* L. (Coleoptera: Coccinellidae), are well-known predators of aphids, and can regulate their population successfully [28]. The larvae of dark soldier beetle, *Cantharis fusca* L. (Coleoptera: Cantharidae), prey primarily on soft-bodied insects, while the food of the imagoes can be insects, besides pollen, nectar, and also honeydew [29]. The woolly aphid parasite, *Aphelinus mali* Hald. (Hymenoptera: Aphelinidae) can regulate the population of its prey species, woolly apple aphid, *Eriosoma lanigerum* Hausm. (Hemiptera: Aphididae) [30]. Predatory mite (Acarina: Phytoseiidae) species can predate spider mites (Acarina: Tetranychidae) effectively [31]. Spiders (Araneae) are generalist predators; i.e., they have a broad prey spectrum. They are very numerous and can provide biocontrol service for agriculture [32].

Only a limited number of studies are available on the varietal preferences of insect pests or natural enemies in apple orchards. Kalinova et al. (2000) [33], Mody et al. (2015) [34], and Stoeva et al. (2025) [35] found that *Anthonomus pomorum* and leafminer moth adults preferred certain apple varieties. Holb et al. (2012) [36] reported that all varieties were strongly damaged by *Cydia pomonella*. The bottom-up effects of apple varieties were detected on the parasitoid wasp, *Ephedrus cerasicola* Stary, *Aphidius matricariae* Hal., and *Aphidius ervi* Hal. (Hymenoptera: Braconidae) via the host species, rosy apple aphid, *Dysaphis plantaginea* (Pass.) (Homoptera: Aphididae) [37,38], as well as on the parasitoids *Scambus pomorum* (Hymenoptera: Ichneumonidae) and *Bracon variator* (Hymenoptera: Braconidae) via *A. pomorum* [39].

Only a few studies are available on the relationship between tree age, canopy structure, and insect damage or natural enemy abundance in apple. Holb et al. (2012) [36] found that tree age had no significant impact on codling moth damage, but it did on the yield. Jacobsen et al. (2022) [40] reported that on older trees, featured with a more complex canopy structure, a more diverse natural enemy community may develop.

Given the scarcity of information on this topic in apple—one of the most important fruit crops worldwide—we aimed to investigate how season, year, orchard structure, and varieties influence the occurrence of insect pest damage and beneficial insects in apple. In addition, we examined the strength of correlations among pest damage, natural enemies, and among different natural enemy taxa.

Variety composition in orchards changes over time in response to market demands and shifting environmental conditions [41,42]. Such changes are both necessary and inevitable and form an integral part of modern production practices. Our objective was therefore to evaluate how these natural changes influence pest damage and the occurrence of beneficial arthropods.

Our hypotheses were as follows:

(1) The abundance of natural enemies and pest damage would differ between and within years.
(2) In Orchard (A), characterized by a larger and more complex leaf canopy structure, insect pest damage would be more severe, and natural enemies (providing an ecosystem service) would be more abundant than in Orchards (B, C), characterized by a smaller canopy.
(3) Apple varieties would influence both the level of arthropod pest damage and the abundance of natural enemy species.

## 2. Materials and Methods

### 2.1. Study Location and Meteorological Data

**Figure 1:**
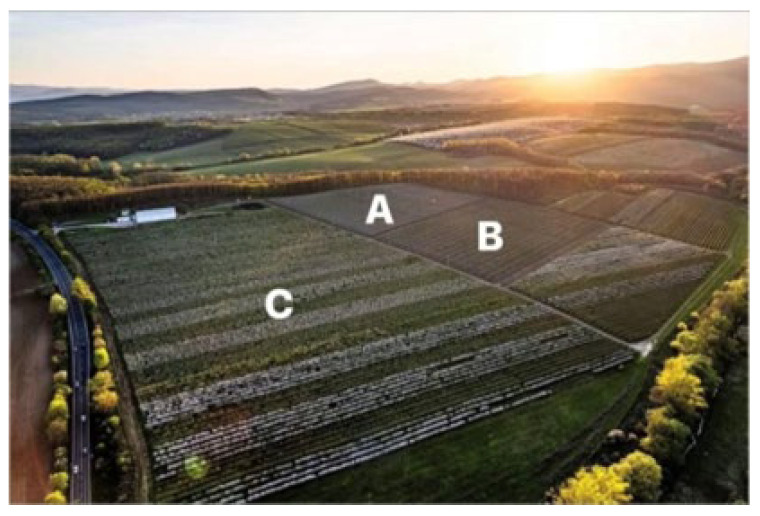
Google Earth photo of the experimental orchards (A: Orchard A; B: Orchard B; C: Orchard C).

Three apple orchards (designated as A, B, and C) were investigated in 2023 and 2024 in Berkenye, northern Hungary. One side of Orchard A was bordered by the main road, another side by Orchards B and C, and the other sides by semi-natural habitats. Two sides of Orchard B were bordered by an oak forest, while the other sides were close to Orchards A and C, respectively (Figure 1). Orchard C was bordered on one side by Orchards A and B and on the other two sides by semi-natural habitats (meadows). The surface area of the adjacent oak forest (*Quercus cerris* L. and *Quercus petraea* (Matt.) Lieb.) was 38.3 ha, while that of the meadows was 5.5 ha. The distance between the semi-natural habitat and the investigated trees ranged from 85 to 300 m in Orchard A. In Orchard B, this distance ranged from 20 to 195 m, and in Orchard C from 20 to 155 m.

At the edge of the oak forest, four woody plant species and seven herbaceous plant species occurred (*Brachypodium sylvaticum* (Huds.) R. et Sch., *Dactylis glomerata* L., *Cornus sanguinea* L., *Crataegus laevigata* (Poir.) DC., *Crataegus monogyna* Jacq., *Festuca heterophylla* Lam., *Ficaria verna* Huds., *Oxalis acetosella* L., *Primula vulgaris* Huds., *Pulmonaria officinalis* L., and *Rosa canina* L.).

In the meadows, six herbaceous plant species were identified (*Dactylis glomerata* L., *Elymus repens* (L.) Gould, *Festuca pratensis* Huds., *Lolium multiflorum* Lam., *Lolium perenne* L., and *Taraxacum officinale* Web. ex. Wigg.).

Although the effect of distance from semi-natural habitats was initially considered, no significant differences were detected for this factor; therefore, this aspect was not included in this paper.

In Orchard A, six weed species dominated both years (*Capsella bursa-pastoris* (L.) Medic., *Conyza canadensis* (L.) Cronq., *Lamium purpureum* L., *Lolium perenne* L., *Portulaca oleracea* L., *Senecio vulgaris* L., and *Taraxacum officinale* Web. ex. Wigg.). In Orchards B and C, in 2023, six weed species occurred most frequently (*Apera spica-venti* (L.) P. B., *Cirsium arvense* (L.) Scop., *Lamium purpureum* L., *Lolium perenne* L., *Stellaria media* (L.) Vill., and *Taraxacum officinale* Web. ex. Wigg.). In 2024, in the two younger orchards, eight weed species occurred predominantly (*Capsella bursa-pastoris* (L.) Medic., *Conyza canadensis* (L.) Cronq., *Elymus repens* (L.) Gould, *Lamium purpureum* L., *Lolium perenne* L., *Senecio vulgaris* L., *Stellaria media* (L.) Vill., and *Taraxacum officinale* Web. ex. Wigg.).

As the manuscript does not focus on weed species, a detailed description of the weed flora was omitted.

All orchards were equipped with drip irrigation. Detailed parameters of the orchards are presented in Table 1.

**Figure 2:**
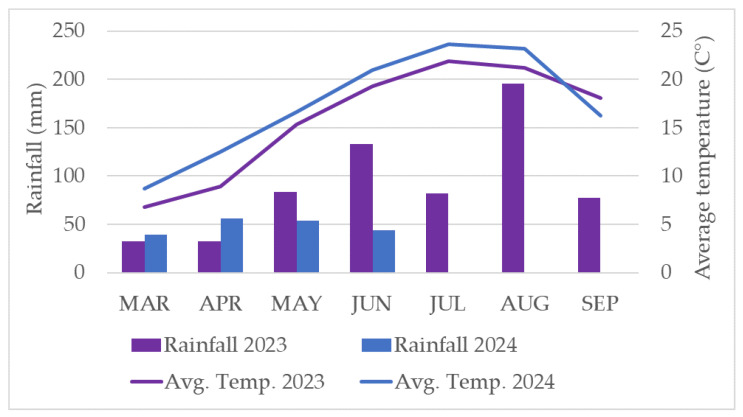
Monthly rainfall and average temperature (Avg. Temp.) data for the vegetation periods 2023 and 2024.

Weather data (daily average temperature and precipitation) were measured at the site of the apple orchards investigated (Berkenye, Hungary) for both vegetation periods (Figure 2).

### 2.2. Parameters of the Investigated Varieties

Galaval Gala: Its tree has a medium growth potential, and its harvest time is in late August. The fruits are sizable, round, crisp, juicy, and sweet, with a dark red cover color and slight striping [43].

Braeburn: It has a weak growth potential; it ripens in October. The fruits are elongated, truncated cone-shaped, and medium to large-sized, with a red color, compact flesh, and harmonic taste [44].

Devil Gala: The tree has a medium growth potential. Fruits are large, with dark red skin, crisp, juicy flesh, and a sweet taste [45].

Gala Must (Regal Prince): It has a medium growth potential, with harvest time beginning in late August. It has medium-large, round, crisp, and sweet fruits with a yellow base color and dark red cover color. It is sensitive to scab [46].

Golden Delicious: Its tree has a medium growth potential. Fruits are medium-large to large, round or slightly elongated, with yellow coloration and middle-firm flesh. It is sweet and mildly subacid. It ripens in September [44].

Golden Reinders: The tree has a medium growth potential, with harvest time in September. It has large fruits with yellow, crisp, and sweet flesh [47].

Idared: It has a medium growth potential, and it ripens in October. It is a medium to large-sized, flat-round, bright red, juicy, and slightly acid apple, with medium-hard flesh [44].

Jonagold: The tree has a strong, late-moderate growth potential; its harvest time is September. Fruits are large and roundish, with a pale red basic color, medium-hard flesh, and sweet-acid taste [44].

Najdared: Its tree has a strong growth potential and ripens in mid-October. Fruits are medium to large, round, with a red cover color and bright skin [48].

Pinova: It has a medium growth potential, and its harvest time is from late September to October. Its fruits are medium to large, conic with a yellow base and red cover color, and sweet taste [49].

Red Delicious: Its tree has a medium or strong growth potential, and it ripens in late September. Fruits are medium to large, elongated, with a dark red color, hard flesh, and sweet taste [46].

Red Jona Prince: The tree has a medium growth potential, and the harvest time is the second half of September. Fruits are large and round, with a bright red cover color and crispy flesh [43].

### 2.3. Pest Management

The three orchards were managed by a single grower. All orchards were managed under an integrated pest management (IPM) system, and no unmanaged or untreated control plots were included. Orchards A, B, and C were managed identically, with pesticide applications carried out on the same dates. Apart from pesticide applications and mechanical weed management, no additional IPM practices were implemented in the investigated orchards. Applications of fungicides, insecticides, and acaricides over the two study years, including their dosages and target organisms, are shown in Tables S1–S4. Chemical and mechanical weed management practices are detailed in Tables S5 and S6. Synthetic herbicides were applied within the tree rows.

### 2.4. Monitoring Methods

For the monitoring of insect pests and natural enemies, ten trees per variety per sector were randomly selected in all the orchards, at a distance of at least 20 m from the orchard edge. The same trees were investigated visually in both vegetation periods, five times a year. Monitoring took place on 13 March, 15 April, 22 May, 11 July, and 8 September in 2023, and 25 March, 15–16 April, 14–15 May, 4–5 July, and 10–11 September in 2024. Fruit damage caused by the codling moth and by the summer fruit tortrix was assessed on 100 apples per tree, and leaf damage caused by the leafminer moths was observed on 100 leaves, while flower damage of the apple blossom weevil was registered on 100 flowers of the investigated trees. The number of natural enemies was assessed on 10 trees per variety per sector, visually. Where there were more sectors per variety (Table 1), the sampling number was multiplied by the number of sectors. All of the independent and dependent variables are presented in Table 2.

### 2.5. Data Preparation and Statistical Analysis

Regarding the preparation for statistical analysis, we conducted a distribution analysis of each instance of pest damage and the number of natural enemies to determine whether the data were normally distributed. We found that the distributions of the pest damage and natural enemy taxa variables examined were right-skewed. In a further step of the data processing, the total number of natural enemies and the number of groups of natural enemies present were calculated for each sampling point (trees).

In addition to this, to quantify the diversity of natural enemies, the Shannon diversity index was calculated for each sampling point using the individual number values of each species and taxa of natural enemies. The index was computed with the following formula:

H′=−∑∑i=1Rpi lnpi

where *R* represents the total number of taxa in a given sampling point and *p_i_* denotes the proportion of individuals attributed to the *i*th taxon to the total number of individuals [50]. We found a right-skewed distribution for the total number of natural enemies, and normal distributions for the number of natural enemy groups and the Shannon diversity index variables.

In the first step of the statistical analysis, the effect of the growing season (2023, 2024), the seasonality (time of sampling in March, April May, July, or September), and the location (Orchards A–C), as fixed effects, was tested by Generalized Linear Mixed Model (GLMM) separately on each pest and NE taxon and the total number of NEs as well as by Multi-way Analysis of Variance (Multi-way ANOVA) on the number of NE groups and the Shannon diversity index of NEs. GLMMs also included the tree ID as a random effect and used gamma regression as the target distribution and relationship with the linear model. Due to the requirement of the models for all positive values, all target (dependent) variable values were transformed using the (x + 1) transformation [51]. Despite the transformations prior to model building and further transformation within the model, the tables presented in the results section contain the mean and standard deviation calculated based on the original, untransformed data. In case of GLMMs, the marginal R-squared values (R^2^_m_) of variables were calculated, while in the case of Multiway ANOVAs, partial eta-squared values (η^2^_p_) were calculated to describe the size effect [52,53]. Significant cases of each test were also tested using Tukey’s post hoc test (for seasonality and plantation) [54].

The second step involved creating a variable using year and seasonality. This produced a variable with ten time-period groups. Due to the large variations between plantations, the effect of this merged variable on pest and NE taxa and the total number of NEs, where data was transformed by ln(x + 1) transformation, and on the number of NE groups and Shannon diversity index of NEs variables were tested separately by one-way ANOVA for each plantation. The Tukey post hoc test was used to compare variable levels in cases where they were found to be significant. The figures in the results section accompanying this analysis show the untransformed mean and standard deviation values, as well as the statistical evaluation based on the transformed data.

In the third step, to demonstrate the effect of the varieties, one-way ANOVA was used to test all pest and NE taxa and the total number of NEs, where data were transformed by ln(x + 1) transformation, and the number of NE groups and the Shannon diversity index of NEs for varieties, regardless of the plantation, separated by sampling years. As in step 2, Tukey’s post hoc test was used to compare variable levels in significant cases, and figures include untransformed mean and standard deviation values.

In the fourth step, we used the Pearson correlation coefficient to assess the strength of the relationships between the relevant pests and NE relations, as well as the relationships between NE groups, using ln(x + 1) transformation on the original data. The following categories were classified based on correlation coefficients: 0–0.19: very weak; 0.20–0.39: weak; 0.40–0.59: moderate; 0.60–0.79: strong; and 0.80–1.00: very strong.

The R-squared values for GLMMs were calculated in the R environment (version 4.5.2, R Core Team, Vienna, Austria) using the “r2glmm” package (version 0.1.4). The other statistical analyses were performed using the IBM SPSS Statistics for Windows software (version: 29.0.2, IBM Corp., Armonk, NY, USA). All tests were conducted at a 95% confidence level. 

## 3. Results

### 3.1. The Effect of Inter-Annual and Intra-Annual Seasonality, and Orchards

Regarding insect damage, significant differences were detected between years, months, and, except for *Cydia pomonella*, between orchards as well. In 2024, the damage from all pests was significantly more severe. Within the year, the attack of *Cydia pomonella*, *Adoxophyes orana*, and *Phyllonorycter* spp. was significantly greater in May–July, while that of *Anthonomus pomorum* was significantly more severe in April. The greatest damage was found in all cases in Orchard A (Table 3).

As for beneficial insects, significant differences were found between orchards and months across all taxa, and, except for *Orius* spp., between years as well. Except for *Coccinella septempunctata* and *Aphelinus mali*, the number of all natural enemies was significantly higher in 2024. Except for Syrphidae, Raphidioptera, *Aphelinus mali*, and predatory mites, the abundance of all taxa was significantly higher in Orchard B (Table 4).

The total number of natural enemies, the number of natural enemy groups, and the Shannon diversity of natural enemies differed significantly between years, months, and orchards too. In 2024, all variables were significantly higher. In Orchard B, the total number of natural enemies and the number of natural enemy groups were significantly the highest (Table 5).

### 3.2. Seasonal Distribution of Insect Pest Damage

**Figure 3:**
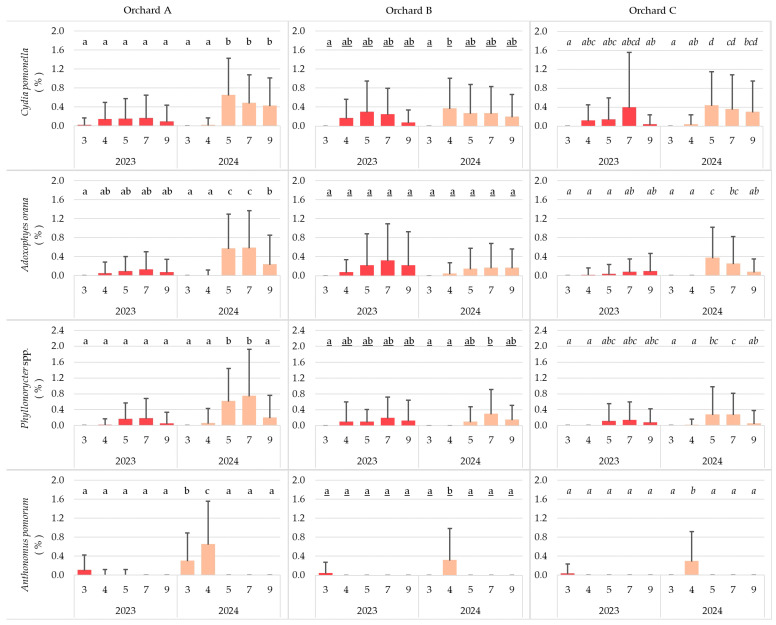
Seasonal distribution of insect pests in the studied orchards (3: March; 4: April; 5: May; 7: July; 9: September) (cases with the same letter mean no significant difference at the 95% confidence level).

The damage caused by *Anthonomus pomorum* could be observed only in spring, in both years, and in all orchards. The other insects attacked the trees primarily in May and July, regardless of year or site. In 2024, the damage caused by all pest species was greater than in 2023, although it was not severe (below 0.8% in any case). In 2023, the damage caused by pest species did not differ significantly among months in any of the three orchards. In contrast, in 2024, significant seasonal differences were detected for all insect taxa. In all orchards, *Cydia pomonella* attacked the most fruits in April or May, depending on the sites. In Orchard A and B, the highest incidence caused by *Adoxophyes orana* and *Phyllonorycter* spp. was observed in July, whereas in Orchard C, peak damage occurred in May (Figure 3).

### 3.3. Seasonal Distribution of Natural Enemies

**Figure 4:**
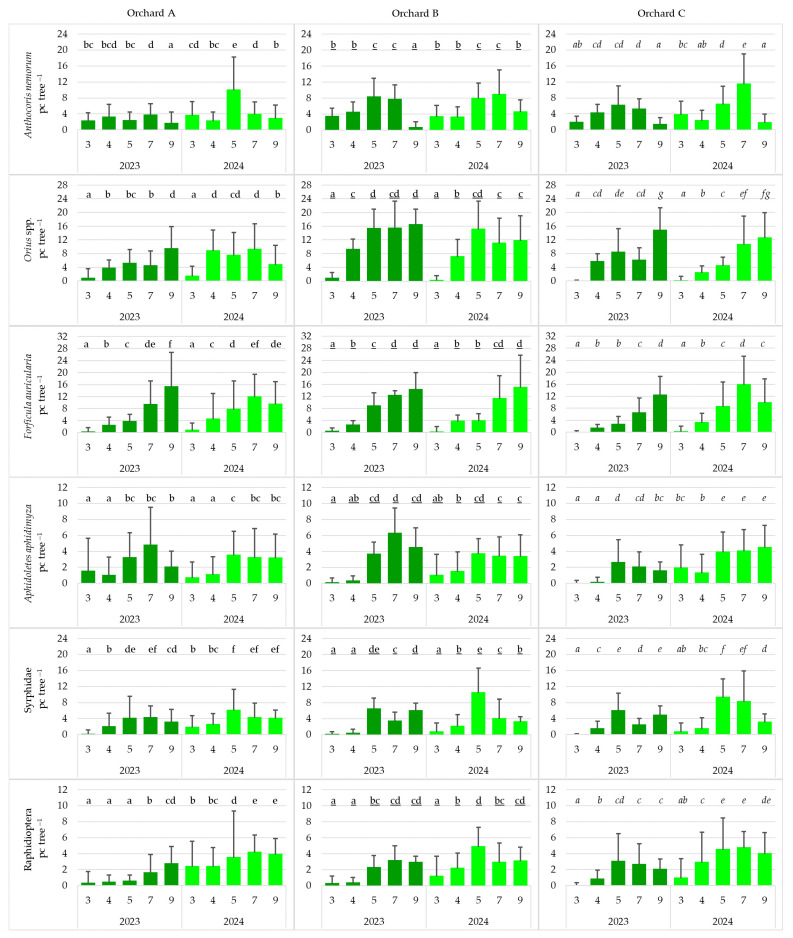

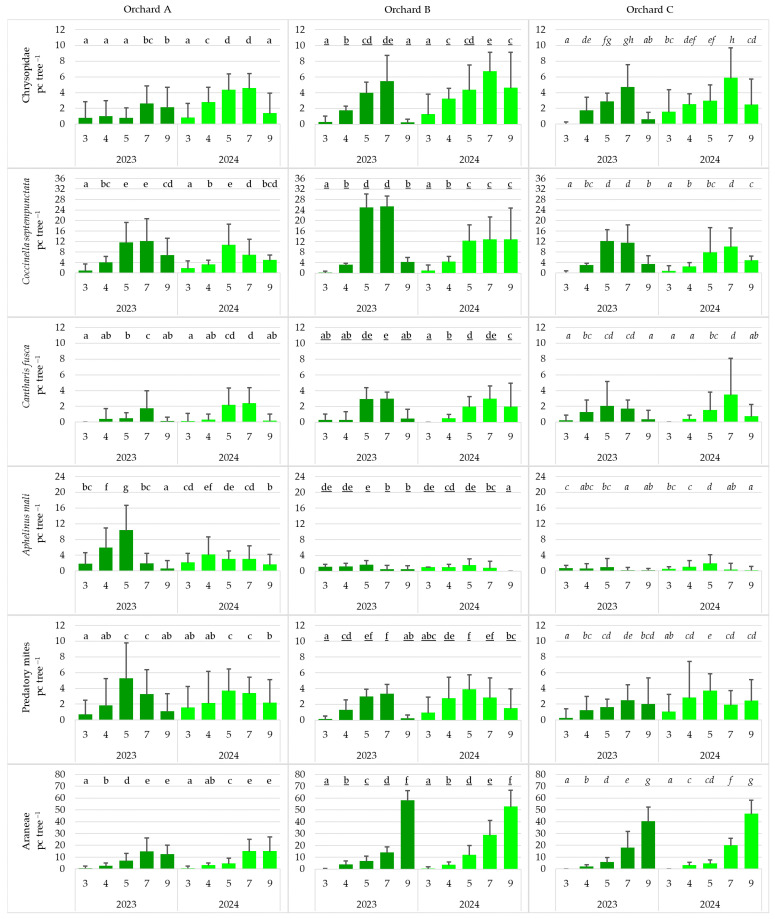
Seasonal distribution of natural enemies in the studied orchards (pc tree^−1^: number of individuals per tree; 3: March; 4: April; 5: May; 7: July; 9: September) (cases with the same letter mean no significant difference at the 95% confidence level).

In both years, and across all orchards, Araneae was the dominant taxon, reaching a maximum abundance of nearly 60 individuals per tree. In contrast, *Aphelinus mali* exhibited the lowest abundance among all taxa, particularly in Orchards B and C, with fewer than four individuals per tree. The abundance of *Forficula auricularia* and Araneae increased progressively during the season in all orchards and years. In 2023, in Orchards A and C, *Orius* spp. and *F. auricularia* were significantly abundant in September. In 2024, in Orchard B, Syrphidae and Chrysopidae were significantly dominant in May. In 2024, the number of *Coccinella septempunctata* was significantly higher in Orchard A in May, whereas in Orchard C, it peaked in July. The abundance of *Cantharis fusca* was significantly the highest in July 2023 in Orchard A and in July 2024 in Orchard C. In Orchard A in 2023, as well as in Orchard C in 2024, *A. mali* was significantly abundant in May. In Orchard B and C, the number of Araneae was significantly the highest in September, in both years (Figure 4).

### 3.4. Seasonal Distribution of Variables Describing the Natural Enemy Populations

**Figure 5:**
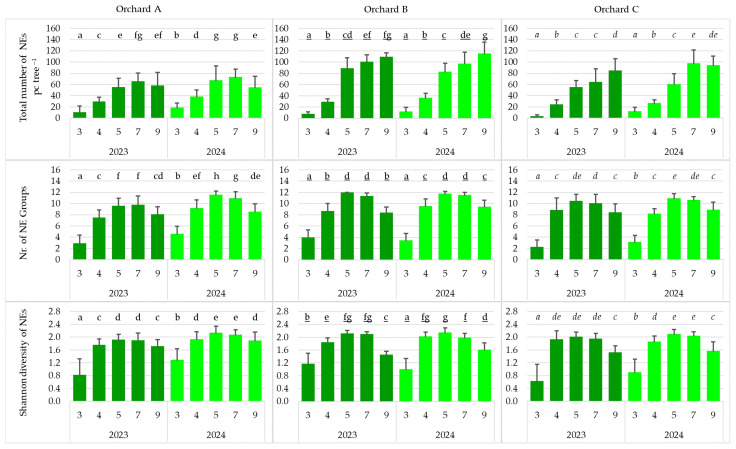
Seasonal distribution of variables describing the natural enemy populations (total number of natural enemies, number of natural enemy groups, Shannon diversity of natural enemies) in the studied orchards (pc tree^−1^: number of individuals per tree; 3: March; 4: April; 5: May; 7: July; 9: September) (cases with the same letter mean no significant difference at the 95% confidence level).

In every orchard and every year, the total number of natural enemies (NEs), the number of natural enemy (NE) groups, and the Shannon diversity of natural enemies increased almost progressively in the vegetation period. In both years and across all orchards, the values of all three variables were significantly lower in March. In Orchard B in 2024, and in Orchard C in 2023, the total number of NEs was significantly higher in September, reaching nearly 120 and over 80 individuals, respectively. In 2024, in Orchard A, the number of NE groups was significantly higher in May (nearly 12) (Figure 5).

### 3.5. Effect of Varieties on Insect Pests

**Figure 6:**
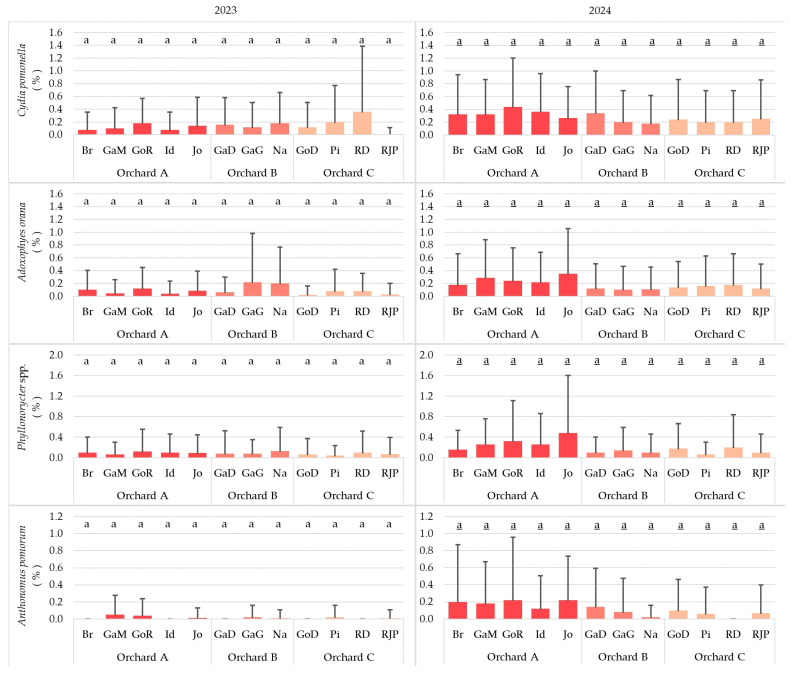
Effect of varieties on the number of insect pests surveyed in 2023 (**left**) and 2024 (**right**) (Br: Braeburn; GaD: Gala Devil; GaG: Gala Galaval; GaM: Gala Must; GoD: Golden Delicious; GoR: Golden Reinders; Id: Idared; Jo: Jonagold; Na: Najdared; Pi: Pinova; RD: Red Delicious; RJP: Red Jona Prince) (cases with the same letter mean no significant difference at the 95% confidence level).

In all the studied apple varieties, insect damage was similar, without any significant difference. However, in 2023, *A. pomorum* proved to be the least harmful to the trees (the damage was 0% or nearly 0%), but in all the orchards in 2024, *C. pomonella* showed itself to be most harmful to all varieties (its damage was mostly between 0.2 and 0.4%). In 2023, the greatest incidence (nearly 0.4%) caused by *C. pomonella* was observed in Orchard C, in the Red Delicious variety, whereas in 2024, its greatest attack was found in Orchard A, in the Golden Reinders variety (above 0.4%). In 2023, the Galaval Gala variety suffered the most severe damage from *A. orana* (0.2%), while in the consecutive year, the most serious damage was recorded in the Jonagold variety (nearly 0.4%). In 2023, *Phyllonorycter* species primarily attacked the leaves of the Najdared variety (0.15%), while in 2024, the most serious incidence was observed on the Jonagold variety (above 0.4%). In 2023, *A. pomorum* was found to be the most harmful to the Gala Must variety (about 0.1%), and in 2024, its damage level was the highest in the varieties Golden Reinders and Jonagold (about 0.2% in both cases) (Figure 6).

### 3.6. Effect of Varieties on Natural Enemies

In 2023, in Orchard C, significant differences among varieties were detected for eight taxa: *A. nemorum*, *Orius* spp., *A. aphidimyza*, Syrphidae, Raphidioptera, *C. fusca*, *A. mali*, and predatory mites. In Orchard B, only the number of *A. aphidimyza* differed significantly between the Gala Galaval and Gala Devil varieties. In Orchard C, there were significant differences among the varieties in the abundance of eight taxa.

**Figure 7:**
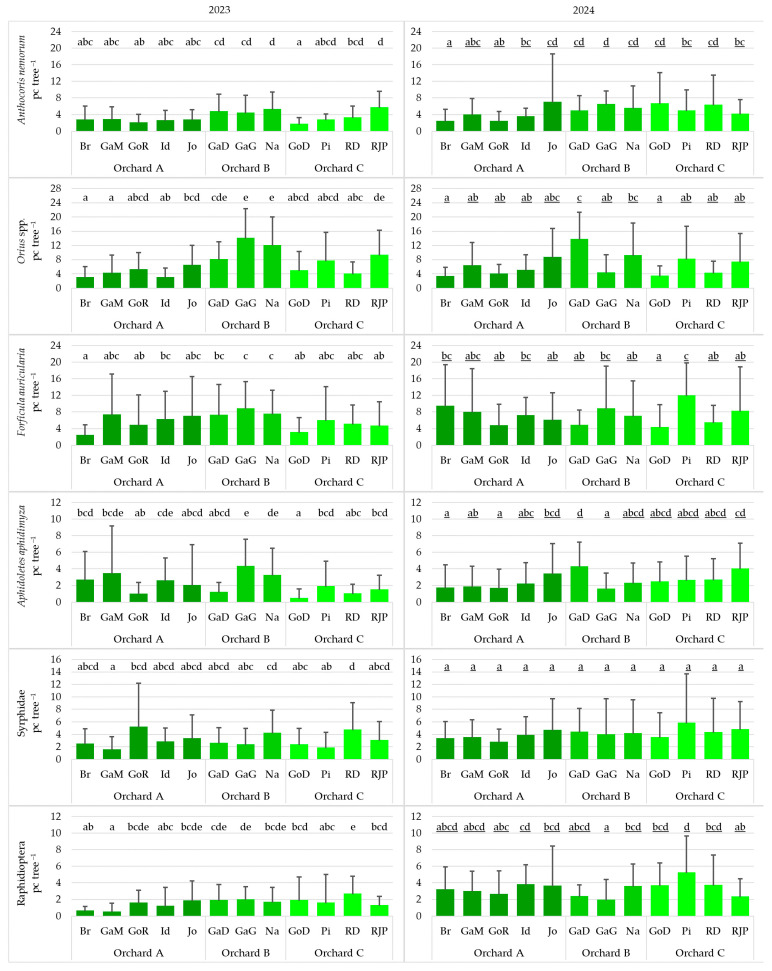

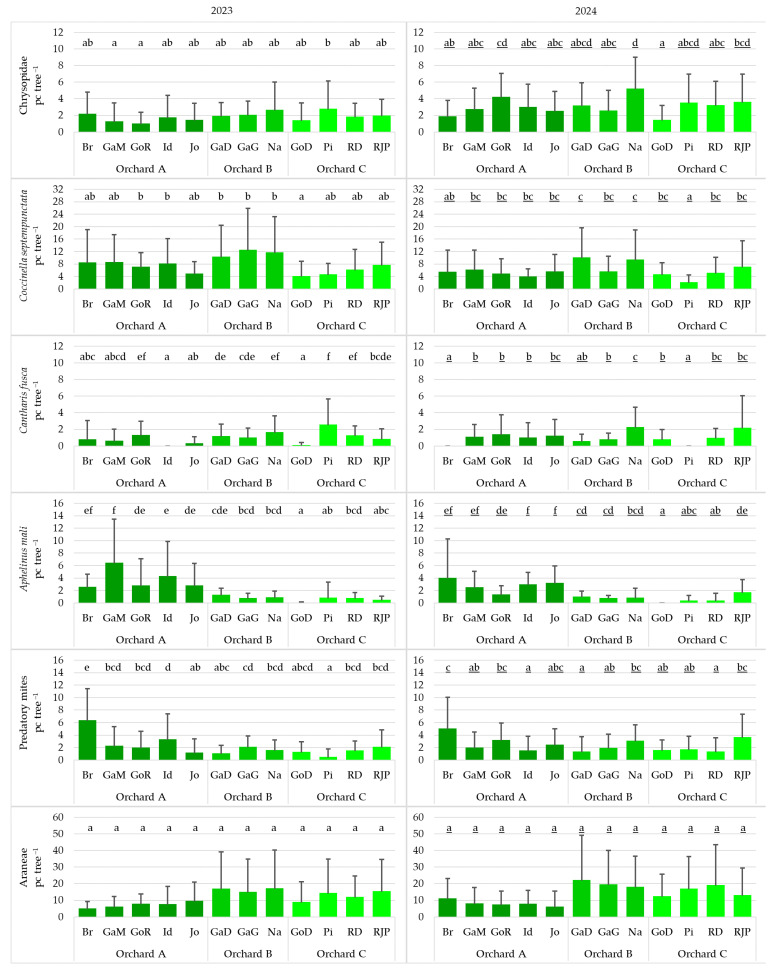
Effect of varieties on the number of natural enemies surveyed in 2023 (**left**) and 2024 (**right**) (pc tree^−1^: number of individuals per tree; Br: Braeburn; GaD: Gala Devil; GaG: Gala Galaval; GaM: Gala Must; GoD: Golden Delicious; GoR: Golden Reinders; Id: Idared; Jo: Jonagold; Na: Najdared; Pi: Pinova; RD: Red Delicious; RJP: Red Jona Prince) (cases with the same letter mean no significant difference at the 95% confidence level).

In 2024, in Orchards A, B, and C, the abundance of seven, six, and seven natural enemy taxa, respectively, differed significantly among varieties (Figure 7).

### 3.7. Effect of Varieties on the Variables Describing the Natural Enemies

**Figure 8:**
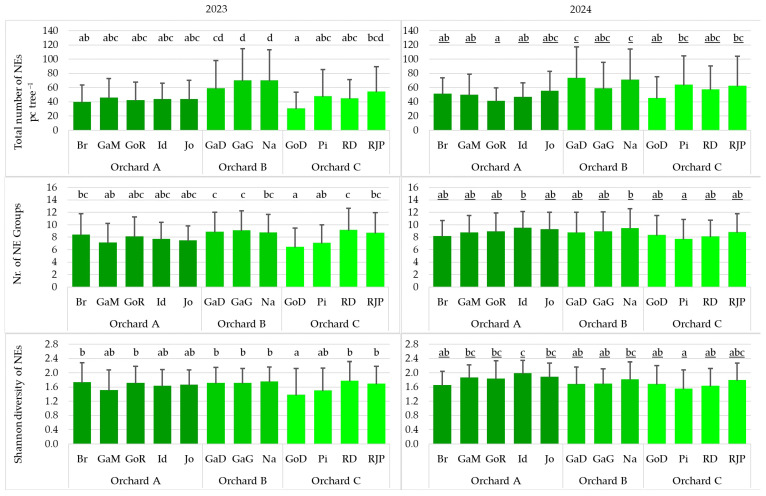
Effect of varieties on variables describing the natural enemy populations in the studied orchards (total number of natural enemies, number of natural enemy groups, Shannon diversity of natural enemies) (pc tree^−1^: number of individuals per tree; Br: Braeburn; GaD: Gala Devil; GaG: Gala Galaval; GaM: Gala Must; GoD: Golden Delicious; GoR: Golden Reinders; Id: Idared; Jo: Jonagold; Na: Najdared; Pi: Pinova; RD: Red Delicious; RJP: Red Jona Prince) (cases with the same letter mean no significant difference at the 95% confidence level).

As for the total number of natural enemies, the number of natural enemy groups, and the Shannon diversity of natural enemies, significant differences among apple varieties were found in only some cases. In 2023, in Orchard C, the values of all three variables were significantly lower in the Golden Delicious trees than in the Red Jona Prince trees. In contrast, in the same year, no significant differences among varieties were detected in Orchards A and B for any of the variables. In 2024, in Orchard A, the Shannon diversity of NEs was significantly higher in the Idared variety than in the Braeburn variety. In the other two orchards, no significant differences were observed among varieties in terms of the total number of NEs, the number of NE groups, or the Shannon diversity (Figure 8).

### 3.8. Correlation Between Pest Damage and Natural Enemies

**Figure 9:**
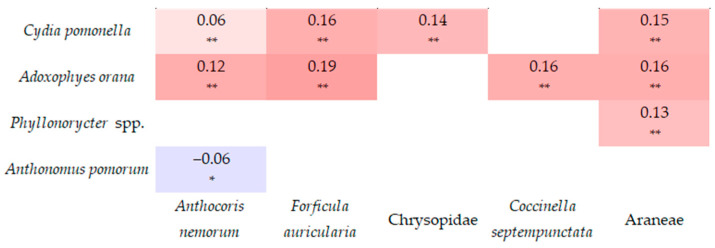
Correlation (Pearson correlation coefficients and significance level) between pests and their relevant natural enemies (* significant on 95% confidence level; ** significant on 99% confidence level).

A very weak (0–0.19) correlation was detected between the damage caused by *C. pomonella* and the number of *F. auricularia* and Chrysopidae, as well as between the damage of *A. orana* and the abundance of *F. auricularia*, *A. nemorum*, *C. septempunctata*, and Araneae (Figure 9).

### 3.9. Correlation Between Natural Enemy Taxa

**Figure 10:**
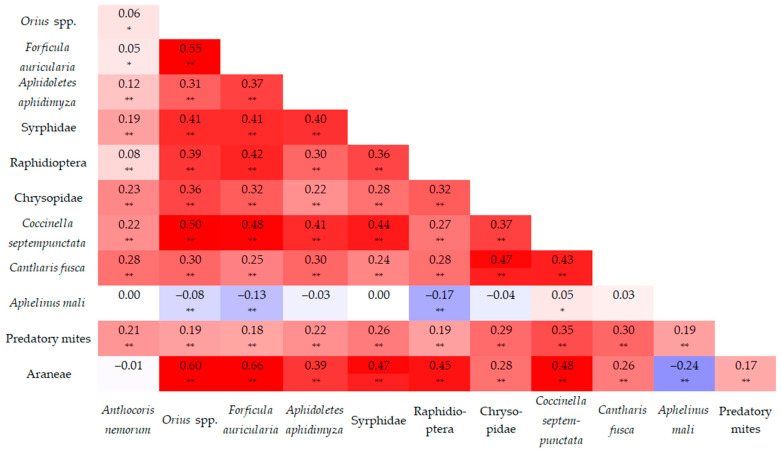
Correlation (Pearson correlation coefficients and significance level) between natural enemy taxa (* significant on 95% confidence level; ** significant on 99% confidence level).

A strong correlation (0.60–0.79) was detected in only two cases: between Araneae and *Orius* spp., and between Araneae and *F. auricularia*. A moderate correlation (0.40–0.59) was observed in fourteen cases, which was similar to the number of very weak correlations (0–0.19). In the remaining cases, correlations were mostly weak (0.20–0.39), occurring in twenty-seven instances (Figure 10).

## 4. Discussion

Sustainable pest management in fruit production faces increasing challenges due to climate change and reduced pesticide availability. Our manuscript provides new insights into the seasonal and interannual variability of insect pests and their natural enemies across apple orchards of different ages and structures in Hungary, one of the most important apple producers in Europe.

According to our results, the main pests were the codling moth (*Cydia pomonella*), the summer fruit tortrix (*Adoxophyes orana*), the leafminer moth (*Phyllonorycter* spp.), and the apple blossom weevil (*Anthonomus pomorum*). No aphid damage was observed in any of the investigated orchards, probably due to the high abundance and variability of aphidophagous insects.

As we hypothesized, the extent of pest damage and the population dynamics of beneficial insects were clearly influenced both seasonally and interannually. In 2024, the damage caused by all insect pests was more severe. These findings correspond with those of Bale et al. (2002) [55] and Deutsch et al. (2008) [56], who emphasized that temperature has a crucial effect on insect development and the number of generations.

The early-season dominance of *Anthonomus pomorum* damage observed in our study corresponds to its univoltine life cycle and the early spring appearance and feeding activity of imagoes, followed by larval damage [57]. The phenology of this species is closely related to the bud sprouting of apple trees [20].

Although *Anthocoris nemorum* was previously reported to dominate in apple orchards in spring [22], our observations revealed that this species was dominant in July 2024 in Orchards B and C.

In the case of pest species, smaller differences were found between orchards. This may be because their population dynamics are more influenced by regional environmental factors than by local plantation parameters [58,59].

In contrast, the abundance of several natural enemy taxa (e.g., *Orius* spp., Chrysopidae, *Cantharis fusca*, Araneae) varied significantly among orchards, demonstrating that these insect groups respond more sensitively to the environmental and biological heterogeneity within the plantations. Previous studies have demonstrated that predator abundance and activity are primarily influenced by prey availability and plant phenology, as these factors determine the accessibility of food and refuge sources. Landis et al. (2000) [60] highlight that the population dynamics of predators and parasitoids follow the seasonal changes in prey availability and vegetation phenology, particularly in perennial crops, such as apple orchards. Similarly, Mezőfi et al. (2020) [61] found that the activity and species richness of arthropod predators in apple orchards vary markedly during the vegetation period, peaking in early spring when prey and habitat conditions are more favorable.

To understand interannual differences in the population dynamics of pest and beneficial insect species, it is crucial to consider the meteorological conditions prevailing during the studied years. Marked differences were observed between the two vegetation periods with respect to both precipitation and average temperature. In 2023, higher rainfall was recorded during the second half of the summer, particularly in August, whereas in 2024, warmer and drier conditions predominated during several months. These deviations may have influenced the development, survival, and population dynamics of arthropods, in accordance with the findings of Bale et al. (2002) [55]. During the first vegetation period, the incidence of *Cydia pomonella*, *Adoxophyes orana*, and *Phyllonorycter* spp. was lower than that observed in 2024. Toward the end of summer, increased precipitation may have promoted fungal infections, while simultaneously reducing imaginal activity (e.g., swarming and mating) [62].

In 2023, the abundance of certain predator taxa, namely Chrysopidae and Syrphidae, was lower than in 2024. This difference may be partly explained by the reduced species richness of available prey. In addition, periods of increased rainfall may negatively affect predator activity on the soil surface or on plants [62].

Insect damage, as well as the abundance of most predator taxa, was higher in 2024. This pattern may be attributed to more favorable temperature conditions in that year, which could enhance arthropod activity, fecundity, and developmental rates [55].

Across all investigated orchards, insecticide applications likely contributed to maintaining pest damage at a low level in both years. However, the management of *C. pomonella* is becoming increasingly difficult as a consequence of climate change, pesticide resistance, and the reduction in approved pesticide registrations [17].

The use of granulovirus- or *Bacillus thuringiensis*-based formulations allowed the beneficial insects to establish themselves and increase in abundance. These selective pesticides are less harmful to the natural enemies [63]. According to our observations, it seems that the applied spray program did not inhibit the presence of predator or parasitoid organisms. Overall, our results demonstrate that the application of integrated pest management (IPM) technology was compatible with the persistence of natural enemies, allowing natural control mechanisms to operate effectively in the studied orchards.

Correlations between the damage of pests and their natural enemies were very weak, in agreement with previous studies. The population of generalist predators often does not directly track pest dynamics, as the availability of alternative prey may weaken their direct interactions [64,65]. Other contributing factors may include intraguild predation or predator interference [66,67], seasonal effects [68], and hunting strategies [69,70]. On the contrary, between beneficial insect taxa, strong (only in two cases), moderate, and very weak, but mostly weak, correlations were found. However, it is important to underline that correlations may largely reflect synchronous seasonal dynamics rather than direct trophic interactions.

Insect pests and their seasonal dynamics can also be affected by plant-mediated interactions [71]. As we hypothesized, our results indicate that apple variety significantly influenced beneficial insect populations; however, herbivore damage also differed among varieties, but not significantly. Similarly, Holb et al. (2012) [36] found no significant differences between the damage caused by *Cydia pomonella* in the investigated apple varieties. In contrast, variety preference for *Anthonomus pomorum* [33,34] and leafminer moth adults [35] has been reported in other studies.

Contrary to our hypothesis, in Orchard A, which had a larger and more complex leaf canopy structure, only the abundance of the parasitic wasp, *Aphelinus mali*, and predatory mites was higher compared to Orchards B and C. Despite its smaller canopy volume, Orchard B supported the highest abundance of natural enemies in many cases, likely due to its proximity to semi-natural habitats, similarly to the findings of Miliczky and Horton (2005) [72].

There are several limitations that may have affected our results.

For example, the uniform application of IPM-based pesticide treatments across orchards may have reduced the detectability of interactions between natural enemies and arthropod pests. Furthermore, conducting monitoring at more frequent intervals would enable a more sophisticated presentation of the results. Examining more orchards with the same variety assortment would be worthwhile.

## 5. Conclusions

Our results demonstrate that the arthropod community of the investigated orchards is regulated by ecological factors. The population dynamics of pest species are primarily influenced by seasonal and temporal environmental variations. In contrast, beneficial insects appear to be shaped more by the characteristics of the local habitat, mainly by the orchard structure.

Orchard age, however, cannot be disentangled from structural and landscape attributes under the present design, and results should be interpreted accordingly.

No effect of the varieties on insect damage could be detected, whereas the abundance of beneficial organisms differed significantly among varieties in several cases.

The weak correlations between pests and their natural enemies may suggest that the presence and activity of predators depend not only on pest abundance but also on other interrelated ecological factors in intensive apple orchards.

Under conditions of low pest pressure and uniformly applied integrated pest management, it becomes challenging to attribute variation in crop damage to the activity of natural enemies or to differences in habitat structure.

## Figures and Tables

**Table 1:** Cultivation technology data and GPS coordinates of the studied orchards.

	Orchard A	Orchard B	Orchard C
Size (hectare)	10	5	4.5
Year of the plantation	1998	2022	2023
Varieties (Number of sectors ^A^)	Braeburn (1), Golden Reinders (1), Gala Must (3), Idared (1), Jonagold (3)	Najdared (2), Galaval Gala (1), Devil Gala (1)	Red Jona Prince (2), Red Delicious (1), Pinova (1)
Cultivation method	Slender spindle	Super spindle	Super spindle
Row and plant spacing	4 × 1.2 m	3.7 × 0.8 m	3.7 × 0.8 m
Hail protection net	No	Yes	Yes
Line spacing	Natural weed cover	Grassed	Grassed
Latitude	47.90236236° N	47.90281748° N	47.90415162° N
Longitude	19.10094946° E	19.09679301° E	19.09797394° E

^A^ Sector: Contains 10–16 rows, depending on the variety.

**Table 2:** Independent and dependent variables of analysis.

Variables (Unit)	Categories/Range
Independent (Input) variables	
Year	2023, 2024
Month	March, April, May, July, September
Plantation	Orchard A; Orchard B; Orchard C
Dependent (Output) variables	
Damage level of pests (%)	
*Cydia pomonella*	0–15
*Adoxophyes orana*	0–4
*Phyllonorycter* spp.	0–25
*Anthonomus pomorum*	0–3
Natural enemy (NE) species and taxa (pc tree^−1^)	
*Anthocoris nemorum*	0–53
*Orius* spp.	0–66
*Forficula auricularia*	0–77
*Aphidoletes aphidimyza*	0–54
Syrphidae	0–33
Raphidioptera	0–54
Chrysopidae	0–14
*Coccinella septempunctata*	0–38
*Cantharis fusca*	0–16
*Aphelinus mali*	0–24
Predatory mites	0–20
Araneae	0–73
Variables describing the NE individual number	
Total number of NEs (pc tree^−1^)	1–157
Number of NE groups	1–12
Shannon diversity of NEs	0–2.44

NE: natural enemy.

**Table 3:** The effect of inter-annual and intra-annual seasonality, as well as orchards, on damage caused by the studied insect pests.

**Factor(s)/** **Levels**	***Cydia pomonella***				***Adoxophyes orana***			
	**F**	**Sig ^A^**	**R^2^_m_**	**Mean ± SD ^B^**	**F**	**Sig ^A^**	**R^2^_m_**	**Mean ± SD ^B^**
Year	27.39	<0.001	0.14		34.3	<0.001	0.1	
2023			0.13 ± 0.62				0.09 ± 0.35	
2024			0.27 ± 0.56				0.21 ± 0.52	
Month	25.34	<0.001	0.23		38.85	<0.001	0.25	
March			0.01 ± 0.07 a				0.00 ± 0.00 a	
April			0.13 ± 0.36 b				0.03 ± 0.18 a	
May			0.35 ± 0.64 c				0.27 ± 0.57 c	
July			0.33 ± 0.96 c				0.28 ± 0.60 c	
September			0.21 ± 0.48 b				0.15 ± 0.46 b	
Orchard	0.59	ns	0.06		6.06	0.002	0.09	
Orchard A			0.22 ± 0.49				0.18 ± 0.48 b	
Orchard B			0.19 ± 0.48				0.14 ± 0.48 ab	
Orchard C			0.18 ± 0.81				0.10 ± 0.35 a	
**Factor(s)/** **Levels**	***Phyllonorycter* spp.**				***Anthonomus pomorum***			
	**F**	**Sig**	**R^2^_m_**	**Mean ± SD ^A^**	**F**	**Sig**	**R^2^_m_**	**Mean ± SD ^A^**
Year	18.54	<0.001	0.1		57.33	<0.001	0.15	
2023			0.09 ± 0.35				0.02 ± 0.13	
2024			0.22 ± 0.98				0.13 ± 0.45	
Month	20.94	<0.001	0.25		50	<0.001	0.27	
March			0.00 ± 0.00 a					0.12 ± 0.36 b
April			0.04 ± 0.36 a				0.24 ± 0.61 c	
May			0.28 ± 0.61 b				0.00 ± 0.05 a	
July			0.35 ± 1.42 b				0.00 ± 0.00 a	
September			0.11 ± 0.42 a				0.00 ± 0.00 a	
Orchard	5.15	0.006	0.11		13.87	<0.001	0.13	
Orchard A			0.21 ± 0.96 b				0.11 ± 0.41 b	
Orchard B			0.11 ± 0.40 ab				0.04 ± 0.24 a	
Orchard C			0.10 ± 0.39 a				0.03 ± 0.22 a	

^A^ ns: not significant on 95% confidence level; ^B^ Damage level of pests (%). Original, untransformed values. Levels of factors with the same letter are not significant at the 95% confidence level.

**Table 4:** The effect of inter-annual and intra-annual seasonality, as well as orchards, on the studied natural enemy populations.

**Factor(s)** **/Levels**	***Anthocoris nemorum***				***Orius*** ** spp.**				***Forficula auricularia***			
	**F**	**Sig ^A^**	**R^2^_m_**	**Mean ± SD ^B^**	**F**	**Sig ^A^**	**R^2^_m_**	**Mean ± SD ^B^**	**F**	**Sig ^A^**	**R^2^_m_**	**Mean ± SD ^B^**
Year	53.89	<0.001	0.14		3.46	ns	0.06		3.54	ns	0.09	
2023			3.59 ± 3.29				7.00 ± 6.64				6.25 ± 7.5	
2024			5.08 ± 6.34				7.02 ± 7.17				7.26 ± 8.28	
Month	80.49	<0.001	0.48		303.80	<0.001	0.73		406.85	<0.001	0.76	
March			3.14 ± 2.63 ab				0.81 ± 2.14 a				0.52 ± 1.55 a	
April			3.28 ± 2.59 b				6.25 ± 4.54 b				3.24 ± 4.65 b	
May			6.78 ± 8.22 c				8.48 ± 7.16 c				6.03 ± 6.35 c	
July			6.20 ± 5.05 c				8.86 ± 7.19 c				11.28 ± 7.46 d	
September			2.28 ± 2.75 a				10.66 ± 7.27 d				12.71 ± 9.27 e	
Orchard	16.84	<0.001	0.23		40.27	<0.001	0.31		6.69	0.001	0.15	
Orchard A			3.72 ± 5.60 a				5.69 ± 5.81 a				6.73 ± 8.43 ab	
Orchard B			5.35 ± 4.27 c				10.43 ± 8.11 c				7.44 ± 7.23 b	
Orchard C			4.62 ± 4.61 b				6.67 ± 6.78 b				6.25 ± 7.44 a	
**Factor(s)** **/Levels**	***Aphidoletes aphidimyza***				**Syrphidae**				**Raphidioptera**			
	**F**	**Sig ^A^**	**R^2^_m_**	**Mean ± SD ^B^**	**F**	**Sig ^A^**	**R^2^_m_**	**Mean ± SD ^B^**	**F**	**Sig ^A^**	**R^2^_m_**	**Mean ± SD ^B^**
Year	9.63	ns	0.12		67.83	<0.001	0.15		283.32	<0.001	0.36	
2023			2.33 ± 3.76				3.03 ± 3.46				1.51 ± 1.97	
2024			2.69 ± 2.89				4.19 ± 4.56				3.28 ± 3.19	
Month	64.75	<0.001	0.56		209.07	<0.001	0.68		83.34	<0.001	0.55	
March			1.01 ± 3.50 a				0.77 ± 1.95 a				1.03 ± 2.27 a	
April			0.98 ± 1.99 a				1.96 ± 2.63 a				1.57 ± 2.22 b	
May			3.48 ± 2.65 bc				6.67 ± 5.24 c				2.93 ± 3.89 c	
July			3.99 ± 4.30 c				4.59 ± 4.30 b				3.21 ± 2.42 c	
September			3.08 ± 2.59 c				4.06 ± 2.41 b				3.24 ± 1.97 c	
Orchard	2.13	ns	0.11		0.07	ns	0.04		2.93	ns	0.09	
Orchard A			2.49 ± 3.89				3.36 ± 3.70				2.26 ± 2.95	
Orchard B			2.85 ± 2.84				3.82 ± 4.23				2.39 ± 2.17	
Orchard C			2.27 ± 2.59				3.89 ± 4.59				2.64 ± 2.95	
**Factor(s)** **/Levels**	**Chrysopidae**				***Coccinella septempunctata***				***Cantharis fusca***			
	**F**	**Sig ^A^**	**R^2^_m_**	**Mean ± SD ^B^**	**F**	**Sig ^A^**	**R^2^_m_**	**Mean ± SD ^B^**	**F**	**Sig ^A^**	**R^2^_m_**	**Mean ± SD ^B^**
Year	2.12	ns	0.30		2.58	ns	0.04		12.14	0.001	0.06	
2023			1.82 ± 2.35				7.86 ± 8.47				0.91 ± 1.62	
2024			3.16 ± 2.95				6.18 ± 6.74				1.21 ± 2.09	
Month	105.78	<0.001	0.61		418.73	<0.001	0.76		149.55	<0.001	0.59	
March			0.81 ± 1.97 a				0.99 ± 2.16 a				0.10 ± 0.59 a	
April			2.11 ± 1.80 b				3.49 ± 1.72 b				0.51 ± 1.05 b	
May			3.03 ± 2.32 c				12.55 ± 8.52 d				1.73 ± 2.06 c	
July			4.63 ± 2.96 d				12.07 ± 8.68 d				2.43 ± 2.44 d	
September			1.87 ± 2.89 b				6.02 ± 5.93 c				0.52 ± 1.45 b	
Orchard	11.86	<0.001	0.21		38.23	<0.001	0.24		26.08	<0.001	0.23	
Orchard A			2.14 ± 2.44 a				6.39 ± 6.59 a				0.82 ± 1.59 a	
Orchard B			3.20 ± 3.18 c				10.14 ± 10.35 b				1.45 ± 1.81 c	
Orchard C			2.56 ± 2.79 b				5.67 ± 6.28 a				1.19 ± 2.29 b	
**Factor(s)** **/Levels**	***Aphelinus mali***				**Predatory mites**				**Araneae**			
	**F**	**Sig ^A^**	**R^2^_m_**	**Mean ± SD ^B^**	**F**	**Sig ^A^**	**R^2^_m_**	**Mean ± SD ^B^**	**F**	**Sig ^A^**	**R^2^_m_**	**Mean ± SD ^B^**
Year	1.16	ns	0.01		28.17	<0.001	0.12		3.32	ns	0.09	
2023			2.45 ± 4.27				2.01 ± 2.88				11.20 ± 15.47	
2024			1.86 ± 2.63				2.50 ± 2.90				12.41 ± 16.22	
Month	92.74	<0.001	0.45		64.42	<0.001	0.47		983.40	<0.001	0.90	
March			1.44 ± 1.92 b				0.88 ± 2.00 a				0.36 ± 1.41 a	
April			3.01 ± 4.05 c				2.02 ± 3.35 b				3.18 ± 2.12 b	
May			4.13 ± 5.10 d				3.76 ± 3.05 d				6.55 ± 5.47 c	
July			1.48 ± 2.53 b				2.98 ± 2.33 c				17.56 ± 11.16 d	
September			0.70 ± 1.78 a				1.63 ± 2.64 b				31.40 ± 21.15 e	
Orchard	280.91	<0.001	0.51		8.46	<0.001	0.05		38.55	<0.001	0.34	
Orchard A			3.51 ± 4.47 b				2.53 ± 3.29 b				7.69 ± 9.10 a	
Orchard B			0.92 ± 1.07 a				1.99 ± 2.14 a				18.12 ± 21.65 c	
Orchard C			0.69 ± 1.43 a				1.97 ± 2.61 a				14.18 ± 17.73 b	

^A^ ns: not significant on 95% confidence level; ^B^ pc tree^−1^ (number of individuals per tree), original, untransformed values. Original, untransformed values. Levels of factors with the same letter are not significant at the 95% confidence level.

**Table 5:** The effect of inter-annual and intra-annual seasonality, as well as orchards, on the variables describing the natural enemy populations (total number of natural enemies, number of natural enemy groups, Shannon diversity of natural enemies).

Factor(s)/ Levels	Total Number of Natural Enemies				Number of Natural Enemy Groups				Shannon Diversity of Natural Enemies			
	F	Sig	R^2^_m_	Mean ± SD ^A B^	F	Sig	η^2^_p_	Mean ± SD ^B^	F	Sig	η^2^_p_	Mean ± SD ^B^
Year	69.32	<0.001	0.20		114.7	<0.001	0.06		80.98	<0.001	0.04	
2023			50.0 ± 33.2				8.00 ± 3.08				1.65 ± 0.51	
2024			56.8 ± 34.3				8.85 ± 2.91				1.79 ± 0.44	
Month	1658	<0.001	0.77		1928	<0.001	0.81		1013	<0.001	0.70	
March			11.9 ± 9.2 a				3.47 ± 1.54 a				0.99 ± 0.48 a	
April			31.6 ± 9.8 b				8.58 ± 1.61 b				1.88 ± 0.21 c	
May			66.1 ± 22.0 c				10.91 ± 1.26 d				2.06 ± 0.18 e	
July			79.3 ± 23.1 d				10.60 ± 1.32 c				2.00 ± 0.18 d	
September			78.2 ± 30.3 d				8.56 ± 1.38 b				1.68 ± 0.27 b	
Orchard	178.03	<0.001	0.46		59.12	<0.001			23.19	<0.001	0.03	
Orchard A			47.3 ± 26.1 a				8.29 ± 2.88 a				1.75 ± 0.46 b	
Orchard B			68.1 ± 42.0 c				9.02 ± 3.08 b				1.75 ± 0.44 b	
Orchard C			52.6 ± 35.8 b				8.20 ± 3.17 a				1.66 ± 0.55 a	

^A^ pc tree^−1^ (number of individuals/tree), original, untransformed values; ^B^ levels of factors with the same letter are not significant at the 95% confidence level.

## Data Availability

Data are contained within the article.

## Supplementary Materials

The following supporting information can be downloaded at https://www.mdpi.com/article/10.3390/insects17030341/s1, Table S1: List of fungicide applications performed in the investigated orchards in 2023; Table S2: List of fungicide applications performed in the investigated orchards in 2024; Table S3: List of insecticide and acaricide applications performed in the investigated orchards in 2023; Table S4: List of insecticide and acaricide applications performed in the investigated orchards in 2024; Table S5: List of herbicide applications and mechanic weed control performed in the investigated orchards in 2023; Table S6: List of herbicide applications and mechanical weed control performed in the investigated orchards in 2024.
