# Supplementary material for: Seasonal and Interannual Variability in the Insect Pest Damage and Beneficial Insect Populations Across Apple Orchards of Different Ages
Source: Insects. 2026 Mar 20;17(3):341. doi: 10.3390/insects17030341 (PMC13026748; doi:10.3390/insects17030341)
Supplement: Supplementary file 1 [file insects-17-00341-s001.zip › insects-4104175-supplementary.pdf]

Table S1. List of fungicide applications performed in the investigated orchards in 2023.

Application date	Fungicide name	Active ingredient(s) (concentration)	Target organization	Dose
13.03.	Microkén	Sulfur (800 g kg^{-1})	*Podosphaera leucotricha*	4.0 kg ha^{-1}
22.03.	Hydrostar	Copper hydroxide (770 g kg^{-1})	*Venturia inaequalis*	2.0 kg ha^{-1}
22.03.	Microkén	Sulfur (800 g kg^{-1})	*Podosphaera leucotricha*	4.0 kg ha^{-1}
29.03.	Alcoban WG	Dithianon (700 g kg^{-1})	*Venturia inaequalis*	0.45 L ha^{-1}
29.03.	Microkén	Sulfur (800 g kg^{-1})	*Podosphaera leucotricha*	4.0 kg ha^{-1}
05.04.	Faban 500 SC	Dithianon (250 g L^{-1}) + Pyrimethanil (250 g L^{-1})	*Venturia inaequalis*	1.2 L ha^{-1}
12.04.	Chorus 50 WG	Cyprodinil (500 g kg^{-1})	*Venturia inaequalis*	0.4 kg ha^{-1}
12.04.	Delan 700 WG	Dithianon (700 g kg^{-1})	*Venturia inaequalis*	0.5 kg ha^{-1}
18.04.	Revyona	Mefentrifluconazole (75 g L^{-1})	*Podosphaera leucotricha*	1.7 L ha^{-1}
18.04.	Delan Pro	Dithianon (125 g L^{-1}) + Potassium phosphonate (561 g L^{-1})	*Venturia inaequalis*	2.5 L ha^{-1}
25.04.	Faban 500 SC	Dithianon (250 g L^{-1}) + Pyrimethanil (250 g L^{-1})	*Podosphaera leucotricha*	1.2 L ha^{-1}
02.05.	Dagonis	Difenoconazole (50 g L^{-1}) + Fluxapyroxad (75 g L^{-1})	*Venturia inaequalis*	1.0 L ha^{-1}
10.05.	Delan 700 WG	Dithianon (700 g kg^{-1})	*Venturia inaequalis*	0.35 kg ha^{-1}
10.05.	Alfa Solo	Difenoconazole (250 g L^{-1})	*Venturia inaequalis*	0.25 L ha^{-1}
15.05.	Revyona	Mefentrifluconazole (75 g L^{-1})	*Venturia inaequalis*	1.8 L ha^{-1}
15.05.	Kaplan 80 WG	Captan 800 (g kg^{-1})	*Venturia inaequalis*	1.9 kg ha^{-1}
22.05.	Alfa Solo	Difenoconazole (250 g L^{-1})	*Venturia inaequalis*	0.25 L ha^{-1}
01.06.	Sercadis	Fluxapyroxad (300 g L^{-1})	*Monilinia fructigena*	0.3 L ha^{-1}
07.06.	Syllit 400 SC	Dodine (400 g L^{-1})	*Venturia inaequalis*	2.25 L ha^{-1}
08.06.	Alfa Solo	Difenoconazole (250 g L^{-1})	*Venturia inaequalis*	0.25 L ha^{-1}
20.06.	Polyram DF	Metiram (700 g kg^{-1})	*Venturia inaequalis*	2.25 kg ha^{-1}
10.07.	Merpan 80 WDG	Captan 800 (g kg^{-1})	*Venturia inaequalis*	2.0 kg ha^{-1}
10.07.	Cyflamid	Cyflufenamid (50 g L^{-1})	*Venturia inaequalis*	0.5 L ha^{-1}
19.07.	Merpan 80 WDG	Captan (800 g kg^{-1})	*Monilinia fructigena*	2.0 kg ha^{-1}
19.07.	Topas 100 EC	Penconazole (100 g L^{-1})	*Venturia inaequalis*	0.5 L ha^{-1}
01.08.	Flint Max	Tebuconazole (500 g L^{-1}) + Trifloxystrobin (250 g L^{-1})	*Venturia inaequalis*	0.3 kg ha^{-1}
08.08.	Luna Experience	Fluopyram (200 g L^{-1}) + Tebuconazole (200 g L^{-1})	*Venturia inaequalis*	0.5 L ha^{-1}
22.11.	Hydrostar	Copper hydroxide (770 g kg^{-1})	*Venturia inaequalis*	2.0 kg ha^{-1}

Table S2. List of fungicide applications performed in the investigated orchards in 2024.

Application date	Fungicide name	Active ingredient(s) (concentration)	Application aim / target organization	Dose
27.02.	Hydrostar	Copper hydroxide (770 g kg^{-1})	Washing spray	2.0 kg ha^{-1}
04.03.	Syllit 400 SC	Dodine (400 g L^{-1})	*Venturia inaequalis*	2.25 L ha^{-1}
08.03.	Faban 500 SC	Dithianon (250 g L^{-1}) + Pyrimethanil (250 g L^{-1})	*Venturia inaequalis*	1.2 L ha^{-1}
08.03.	Microthiol Max	Sulfur (800 g kg^{-1})	*Podosphaera leucotricha*	5.0 kg ha^{-1}
12.03.	Chorus 50 WG	Cyprodinil (500 g kg^{-1})	*Venturia inaequalis*	0.4 kg ha^{-1}
12.03.	Delan 700 WG	Dithianon (700 g kg^{-1})	*Venturia inaequalis*	0.45 kg ha^{-1}
15.03.	Faban 500 SC	Dithianon (250 g L^{-1}) + Pyrimethanil (250 g L^{-1})	*Venturia inaequalis*	1.2 L ha^{-1}
21.03.	Delan Pro	Dithianon (125 g L^{-1})+ Potassium phosphonate (561 g L^{-1})	*Venturia inaequalis*	2.5 L ha^{-1}
21.03.	Microthiol Max	Sulfur (800 g kg^{-1})	*Podosphaera leucotricha*	5.0 kg ha^{-1}
29.03.	Faban 500 SC	Dithianon (250 g L^{-1}) + Pyrimethanil (250 g L^{-1})	*Venturia inaequalis*	1.2 L ha^{-1}
29.03.	Microkén	Sulfur (800 g kg^{-1})	*Podosphaera leucotricha*	3.0 kg ha^{-1}
04.04.	Chorus 50 WG	Cyprodinil (500 g kg^{-1})	*Venturia inaequalis*	0.4 kg ha^{-1}
04.04.	Target 80 WDG	Captan (800 g kg^{-1})	*Venturia inaequalis*	1.9 kg ha^{-1}
12/04	Dagonis	Difenoconazole (50 g L^{-1}) + Fluxapyroxad (75 g L^{-1})	*Venturia inaequalis*	1.0 L ha^{-1}
12.04.	Target 80 WDG	Captan (800 g kg^{-1})	*Venturia inaequalis*	1.9 kg ha^{-1}
15.04.	Delan Pro	Dithianon (125 g L^{-1})+ Potassium phosphonate (561 g L^{-1})	*Venturia inaequalis*	2.5 L ha^{-1}
15.04.	Chorus 50 WG	Cyprodinil (500 g kg^{-1})	*Venturia inaequalis*	0.4 kg ha^{-1}
17.04.	Syllit 400 SC	Dodine (400 g L^{-1})	*Podosphaera leucotricha*	2.25 L ha^{-1}
22.04.	Faban 500 SC	Dithianon (250 g L^{-1}) + Pyrimethanil (250 g L^{-1})	*Venturia inaequalis*	1.2 L ha^{-1}
22.04.	Cyflamid	Cyflufenamid (50 g L^{-1})	*Podosphaera leucotricha*	0.4 L ha^{-1}
06.05.	Merpan 80 WDG	Captan (800 g kg^{-1})	*Monilinia fructigena*	1.9 kg ha^{-1}
06.05.	Score 250 EC	Difenoconazole(250 g L^{-1})	*Venturia inaequalis*	0.25 L ha^{-1}
06.05.	Cidely	Cyflufenamid (50 g L^{-1})	*Podosphaera leucotricha*	0.4 L ha^{-1}
10.05.	Delan Pro	Dithianon (125 g L^{-1})+ Potassium phosphonate (561 g L^{-1})	*Venturia inaequalis*	2.5 L ha^{-1}
10.05.	Score 250 EC	Difenoconazole (250 g L^{-1})	*Venturia inaequalis*	0.25 L ha^{-1}
14.05	Syllit 400 SC	Dodine (400 g L^{-1})	*Venturia inaequalis*	2.25 L ha^{-1}
16.05	Luna Experience	Fluopyram (200 g L^{-1}) + Tebuconazole (200 g L^{-1})	*Venturia inaequalis*	0.5 L ha^{-1}
16.05	Target 80 WDG	Captan (800 g kg^{-1})	*Monilinia fructigena*	2.0 kg ha^{-1}
23.05	Alfa Solo	Difenoconazole(250 g L^{-1})	*Monilinia fructigena*	0.25 L ha^{-1}
23.05	Target 80 WDG	Captan (800 g kg^{-1})	*Venturia inaequalis*	2.0 kg ha^{-1}
03.06	Sercadis	Fluxapyroxad (300 g L^{-1})	*Venturia inaequalis*	0.3 L ha^{-1}
11.06	Delan Pro	Dithianon (125 g L^{-1})+ Potassium phosphonate (561 g L^{-1})	*Venturia inaequalis*	2.0 L ha^{-1}
11.06	Revyona	Mefentrifluconazole (75 g L^{-1})	*Venturia inaequalis*	2.0 L ha^{-1}

24.06	Target 80 WDG	Captan (800 g kg^{-1})	*Venturia inaequalis*	1.8 kg ha^{-1}
24.06	Topas 100 EC	Penconazole (100 g L^{-1})	*Podosphaera leucotricha*	0.25 L ha^{-1}
08.07	Target 80 WDG	Captan (800 g kg^{-1})	*Venturia inaequalis*	1.9 kg ha^{-1}
22.07	Flint Max	Tebuconazole (500 g kg^{-1})+ Trifloxystrobin (250 g kg^{-1})	*Venturia inaequalis*	0.3 kg ha^{-1}
05.08	Luna Experience	Fluopyram (200 g L^{-1}) + Tebuconazole (200 g L^{-1})	*Venturia inaequalis*	0.5 L ha^{-1}
04.09	Bellis	Boscalid (252 g kg^{-1}) + Pyraclostrobin (128 g kg^{-1})	*Venturia inaequalis*	0.8 kg ha^{-1}
12.09	Bellis	Boscalid (252 g kg^{-1})+ Pyraclostrobin (128 g kg^{-1})	*Venturia inaequalis*	0.8 kg ha^{-1}

Table S3. List of insecticide and acaricide applications performed in the investigated orchards in 2023.

Application date	Insecticide / acaricide name	Active ingredient(s) (concentration)	Application aim / target organization	Dose
13.03.	Catane	Paraffin oils (795 g L^{-1})	Washing spray	25.0 L ha^{-1}
02.05.	Klartan 24 EW	Tau–fluvalinate (240 g L^{-1})	*Cydia pomonella*	0.3 L ha^{-1}
22.05.	Coragen 20 SC	Chlorantraniliprole (200 g L^{-1})	*Cydia pomonella*	0.15 L ha^{-1}
22.05.	Sivanto Prime	Flupyradifurone (200 g L^{-1})	Aphidoidea	0.4 L ha^{-1}
01.06.	Pirimor 50 WG	Pirimicarb (500 g kg^{-1})	Aphidoidea	0.5 kg ha^{-1}
08.06.	Coragen 20 SC	Chlorantraniliprole (200 g L^{-1})	*Cydia pomonella*	0.15 L ha^{-1}
20.06.	Mospilan 20 SG	Acetamiprid (200 g kg^{-1})	Aphidoidea	0.125 kg ha^{-1}
10.07.	Madex Twin	Cydia pomonella granulovirus (CpGV) (520 g L^{-1})	*Cydia pomonella*	0.1 L ha^{-1}
16.07.	Pirimor 50 WG	Pirimicarb (500 g kg^{-1})	*Cydia pomonella*	0.5 kg ha^{-1}
01.08.	Carpovirusine biológiai rovarölő szer	Cydia pomonella granulovirus (CpGV) (10^{13} pc L^{-1})	*Cydia pomonella*	1.0 L ha^{-1}
01.08.	Ortus 5 SC	Fenpyroximate (50 g L^{-1})	Acarina	1.0 L ha^{-1}
01.08.	Mospilan 20 SG	Acetamiprid (200 g kg^{-1})	*Cydia pomonella*	0.125 kg ha^{-1}
08.08.	DiPel DF	Bacillus thuringiensis subsp. Kurstaki (540 g kg^{-1})	*Cydia pomonella*	1.5 kg ha^{-1}

Table S4. List of insecticide and acaricide applications performed in the investigated orchards in 2024.

Application date	Insecticide / acaricide name	Active ingredient(s) (concentration)	Application aim / target organization	Dose
27.02.	Catane	Paraffin oils (795 g L^{-1})	Washing spray	25.0 L ha^{-1}
15.04.	Klartan 24 EW	Tau–fluvalinate (240 g L^{-1})	*Cydia pomonella*	0.3 L ha^{-1}
06.05.	Voliam	Chlorantraniliprole (200 g L^{-1})	*Cydia pomonella*	0.15 mL ha^{-1}
10.05.	Pirimor 50 WG	Pirimicarb (500 g kg^{-1})	Aphidoidea	0.5 kg ha^{-1}
16.05.	Mospilan 20 SG	Acetamiprid (200 g kg^{-1})	*Cydia pomonella*	0.25 kg ha^{-1}
23.05.	Coragen 20 SC	Chlorantraniliprole (200 g L^{-1})	*Cydia pomonella*	0.15 L ha^{-1}
03.06.	Teppeki	Flonicamid (500 g kg^{-1})	Aphidoidea	0.14 kg ha^{-1}
11.06.	Carpovirusine biológiai rovarölő szer	Cydia pomonella granulovirus (CpGV) (10^{13} pc L^{-1})	*Cydia pomonella*	1.0 L ha^{-1}

24.06.	Pirimor 50 WG	Pirimicarb 500 (g kg^{-1})	Aphidoidea	0.5 kg ha^{-1}
24.06.	Flumite 200	Flufenzine (diflovidazine) (200 g L^{-1})	Acarina	0.5 L ha^{-1}
08.07.	Mospilan 20 SG	Acetamiprid (200 g kg^{-1})	*Cydia pomonella*	0.25 kg ha^{-1}
22.07.	Carpovirusine biológiai rovarölő szer	Cydia pomonella granulovirus (CpGV) (10^{13} pc L^{-1})	*Cydia pomonella*	1.0 L ha^{-1}
05.08.	Ortus 5 SC	Fenpyroximate (50 g L^{-1})	Acarina	1.0 L ha^{-1}
05.08.	Teppeki	Flonicamid 500 (g kg^{-1})	Aphidoidea	0.14 kg ha^{-1}

Table S5. List of herbicide applications and mechanical weed control performed in the investigated orchards in 2023.

Application date	Herbicide name / control method	Active ingredient(s) (concentration)	Application aim / target organization	Dose
03.05.	Buldozer	Glyphosate 360 (g L^{-1})	Dicotyledonous weeds	5.0 L ha^{-1}
03.05.	Sharpen 330 EC	Pendimethalin (330 g L^{-1})	Dicotyledonous weeds	5.0 L ha^{-1}
19.05.	Mechanical weed control	–	All weeds	–
21.06.	Mechanical weed control	–	All weeds	–
29.07.	Mechanical weed control	–	All weeds	–
26.08.	Mechanical weed control	–	All weeds	–

Table S6. List of herbicide applications and mechanical weed control performed in the investigated orchards in 2024.

Application date	Herbicide name / control method	Active ingredient(s) (concentration)	Application aim / target organization	Dose
12.04.	Buldozer	Glyphosate (360 g L^{-1})	Dicotyledonous weeds	5.0 L ha^{-1}
12.04.	Sharpen 330 EC	Pendimethalin (330 g L^{-1})	Dicotyledonous weeds	5.0 L ha^{-1}
21.05.	Mechanical weed control	–	All weeds	–
10.06.	Mechanical weed control	–	All weeds	–
04.07.	Mechanical weed control	–	All weeds	–
